# Pro- and Anti-Inflammatory Dietary Patterns and Lifestyle Factors Associated with Gastroesophageal Reflux Symptoms in Romanian Adults: A Cross-Sectional Study

**DOI:** 10.3390/nu18081308

**Published:** 2026-04-21

**Authors:** Nina Ciuciuc, Rodica Ana Ungur, Alexandra-Ioana Roșioară, Monica Popa, Dana Manuela Sîrbu, Daniela Curșeu, Codruța Alina Popescu, Iulia Szerasz, Bogdana Adriana Năsui

**Affiliations:** 1 Department of Community Medicine, “Iuliu Hațieganu” University of Medicine and Pharmacy, 400349 Cluj-Napoca, Romania; nina.ciuciuc@umfcluj.ro (N.C.); alexandra.rosioara@umfcluj.ro (A.-I.R.); monica.popa@umfcluj.ro (M.P.); dsirbu@umfcluj.ro (D.M.S.); dcurseu@umfcluj.ro (D.C.); adriana.nasui@umfcluj.ro (B. A.N.); 2 Research Center in Preventive Medicine, Health Promotion and Sustainable Development, “Iuliu Hațieganu” University of Medicine and Pharmacy, 400349 Cluj-Napoca, Romania; 3 Department of Medical Specialties, Faculty of Medicine, “Iuliu Hațieganu” University of Medicine and Pharmacy, 8 Victor Babeș Street, 400012 Cluj-Napoca, Romania; 4 Department of Practical Abilities—Human Sciences, “Iuliu Hațieganu” University of Medicine and Pharmacy, 6 Louis Pasteur Street, 400349 Cluj-Napoca, Romania; cpopescu@umfcluj.ro; 5 Clinical Rehabilitation Hospital, 400335 Cluj-Napoca, Romania; iuliaszerasz@gmail.com

**Keywords:** gastroesophageal reflux disease, dietary patterns, pro-inflammatory diet, lifestyle factors, meal timing

## Abstract

**Background**: Gastroesophageal reflux disease (GERD) is a common digestive disorder with a substantial impact on quality of life. Emerging evidence suggests that dietary patterns and lifestyle behaviors are associated with the occurrence and severity of GERD symptoms; however, integrated data from Romania remain limited. **Objective**: The aim of this study was to evaluate associations between pro- and anti-inflammatory dietary patterns, lifestyle-related behavioral factors, and the presence and severity of gastroesophageal reflux symptoms in an adult Romanian population. **Methods**: A national cross-sectional observational study was conducted using a self-administered online questionnaire. All participants included in the study reported a prior diagnosis of gastroesophageal reflux disease (GERD), and participant classification was based exclusively on current symptomatology assessed using the GERD-Q score. Therefore, comparisons were not performed between patients and a healthy population, but rather between individuals at different stages of clinical expression of the same condition, characterized by a fluctuating course. The instrument included standardized GERD-Q items for symptom assessment, together with questions regarding dietary intake and lifestyle behaviors. Pro-inflammatory (PRO), anti-inflammatory (ANTI), and combined (PRO–ANTI) dietary scores were established. Statistical analyses included comparative and correlational tests as well as multivariable logistic regression models. **Results**: Among the 340 participants included in the study, 72.4% reported symptoms consistent with GERD according to the GERD-Q score. A higher pro-inflammatory dietary score was significantly associated with GERD, with participants in the highest PRO category showing more than a fourfold higher likelihood of GERD in multivariable analyses. Consumption of spicy foods and carbonated beverages was associated with an increased risk of GERD in univariate analyses; however, these associations did not remain significant in multivariable models. Late meals (defined as consumption of one’s last meal of the day less than two hours before bedtime) were independently associated with GERD. Combined analyses indicated a higher risk among participants who reported eating late meals, particularly when combined with large evening meals. Most foods considered protective, along with classical lifestyle factors (smoking, alcohol consumption, and sleeping position), were not independently associated with GERD. **Conclusions**: These findings suggest that overall dietary patterns with pro-inflammatory potential and meal timing in relation to the sleep–wake cycle may be more consistently associated with GERD symptoms in this sample than isolated food items or traditional lifestyle risk factors. Nutritional and behavioral interventions focused on improving overall dietary patterns and avoiding late meals may represent potential strategies for GERD management.

## 1. Introduction

Gastroesophageal reflux disease (GERD) is one of the most common chronic digestive disorders worldwide, with an estimated prevalence of 14% and 20% in the adult population [1,2,3]. According to the Montreal consensus, GERD is a condition in which the reflux of gastric contents causes troublesome symptoms and/or complications, encompassing both esophageal and extraesophageal manifestations [4]. Subsequently, the Lyon consensus further refined the diagnostic criteria by integrating functional investigations such as combined pH–impedance monitoring [5].

In Central and Eastern Europe, the prevalence of GERD has been reported to be increasing over time, with considerable regional variation [2,3].

In Romania, epidemiological data on gastroesophageal reflux disease remain limited and derive from studies employing heterogeneous methodologies. In a clinical study conducted on adults invited to primary-care settings and assessed using the Montreal criteria, GERD was reported in 31% of the participants [6]. In contrast, a regional population-based study conducted in southwestern Romania, based on self-reported troublesome symptoms, reported a prevalence of 17% [7].

Methodological differences and regional particularities related to diet and lifestyle may partly explain the observed variability between these estimates. At present, no recent national studies have comprehensively evaluated the prevalence of GERD and its associated behavioral factors in Romania.

The clinical presentation of GERD is dominated by heartburn and acid regurgitation, often accompanied by epigastric pain, nausea, and sleep disturbances; extraesophageal manifestations have been reported in approximately 15–20% of cases [8].

In Romanian clinical practice, the complexity of GERD manifestations, including extraesophageal symptoms, is acknowledged in recent national clinical guidelines, which emphasize the need for an integrated approach to management [9].

In recent years, the literature has increasingly highlighted the role of dietary inflammatory load in the pathophysiology of GERD. Pro-inflammatory diets—characterized by a high intake of saturated fats, refined sugars, and ultra-processed foods—may promote reflux through multiple mechanisms, including delayed gastric emptying, increased intra-abdominal pressure, and reduced lower-esophageal sphincter tone. Conversely, anti-inflammatory dietary patterns rich in fiber, antioxidants, omega-3 fatty acids, and polyphenols may reduce oxidative stress and mucosal inflammation, thereby exerting a protective effect on the upper gastrointestinal tract [10,11]. Recent evidence increasingly emphasizes the role of overall dietary patterns, rather than isolated food items, in the development of GERD symptoms.

Previous studies have reported heterogeneous and sometimes inconsistent findings regarding the role of individual dietary factors and lifestyle behaviors in GERD, highlighting the need for more integrated approaches.

Behavioral factors such as smoking, alcohol consumption, late and large evening meals, sleeping position, and physical inactivity may further contribute to the severity of GERD symptoms, particularly when combined with unfavorable dietary patterns [10].

In the Romanian context, current nutritional trends—characterized by high consumption of fried food, saturated fats, and sugar-sweetened beverages—are frequently associated with smoking and sedentary lifestyles, indicating a potentially increased risk profile for GERD. However, contemporary national data examining the relationship between diet, lifestyle, and GERD are insufficient.

Lifestyle modifications, particularly weight reduction, have been shown to be effective in alleviating GERD symptoms, as demonstrated by prospective population-based studies [12].

In both clinical practice and population-based observational studies, the GERD-Q questionnaire represents a validated and standardized instrument for assessing typical symptoms and their impact on quality of life [13].

Integrating data on diet, smoking status, alcohol consumption, and symptom frequency may provide a more comprehensive perspective on modifiable risk factors and potential preventive strategies. Although numerous studies have investigated the relationship between dietary habits and GERD, relatively few have simultaneously examined pro- and anti-inflammatory food consumption, behavioral risk factors (smoking and alcohol consumption), eating habits, and GERD symptomatology within a single population [10,14]. However, existing studies have primarily focused on isolated dietary factors or specific behaviors, while integrated analyses simultaneously evaluating pro- and anti-inflammatory dietary patterns together with behavioral factors remain limited, particularly in Eastern European populations. Moreover, few studies have concurrently assessed dietary inflammatory profiles, eating behaviors, and lifestyle-related factors within a unified analytical framework.

To date, the Romanian scientific literature does not include studies that concurrently assess pro- and anti-inflammatory dietary patterns, lifestyle behaviors, and the severity of gastroesophageal reflux symptoms in an adult population. This lack of an integrated approach limits our understanding of the relationship between diet, behavioral factors, and the clinical manifestations of GERD in Romanian adults.

Previous studies have reported GERD prevalence estimates ranging between 14–20% globally and approximately 17% in Romania. However, online survey-based studies may overestimate GERD prevalence due to self-selection of symptomatic individuals.

Given the inherently fluctuating clinical expression of GERD, characterized by alternating periods of symptom presence and remission, evaluating the current symptom status in individuals with a prior diagnosis may provide additional insights into factors associated with symptom variability.

In this context, an integrated evaluation of dietary patterns and associated behaviors may contribute to a better understanding of modifiable factors involved in GERD symptomatology.

Therefore, the aim of this study was to evaluate the associations between dietary patterns with pro- and anti-inflammatory potential, lifestyle-related behavioral factors, and the presence and severity of gastroesophageal reflux symptoms in an adult Romanian population, using an integrated analytical approach.

## 2. Materials and Methods

### 2.1. Study Design and Participants

A cross-sectional study was conducted using a self-administered online questionnaire distributed to adults (≥18 years) residing in Romania.

All participants reported a prior diagnosis of GERD; however, symptom status varied at the time of questionnaire completion. Therefore, participants were classified strictly based on their current symptom status using the GERD-Q score (≥8 vs. <8), while all had a prior diagnosis of GERD. This approach does not represent a case–control design, but rather a comparison between participants at different stages of symptomatic expression within the same underlying condition.

Data were collected online via the Google Forms platform between December 2025 and January 2026. All participants were informed about the purpose of the study and provided informed consent through the voluntary and anonymous completion of the questionnaire; no separate written consent was required due to the anonymous online format.

The online questionnaire platform did not collect identifiable personal data. IP addresses were not stored or accessible to the researchers, ensuring the anonymity of the responses. Additionally, duplicate responses were minimized through questionnaire settings restricting multiple submissions from the same device/session; however, complete elimination of duplicate entries cannot be fully guaranteed in anonymous online surveys.

### 2.2. Recruitment Strategy and Participant Flow

Participants were recruited by distributing the questionnaire through relevant support groups and community pages on social media platforms. A convenience sampling strategy was employed in the online environment. To ensure methodological transparency and justify the final sample size, the theoretical participant flow was reconstructed from the estimated accessible population to obtain the desired final number of valid questionnaires for analysis.

The participant inclusion process followed five sequential stages:

1. Potentially affected population: In Romania, a local study reported a GERD prevalence of 17% [8], which suggests an estimated 2–2.4 million affected individuals when applied to the adult population (~12 million). Based on this prevalence and the level of engagement in online support networks and social media communities, it was estimated that approximately 50,000 individuals with GERD in Romania could be accessible for an online survey.
2. Survey invitation: It was estimated that approximately 10% of these individuals actually viewed the survey invitation, resulting in an estimated 5000 people.
3. Access to the questionnaire link: Applying link access rates reported in the literature for online surveys (~20–25%), we estimated that 1120 individuals would access the questionnaire.
4. Completed questionnaires: In online surveys, the proportion of completed questionnaires is typically reported to range between 35% and 45%. Applying an approximate completion rate of 40% resulted in a theoretical estimate of 448 completed questionnaires, which is close to the actual number of responses obtained (n = 412).
5. Inclusion/exclusion criteria: Application of the inclusion criteria (age ≥ 18 years, residence in Romania, fully completed questionnaire, and valid responses to GERD-Q items) led to the exclusion of 72 questionnaires, resulting in 340 valid questionnaires, which constituted the final dataset for statistical analysis. The exclusion criteria included age < 18 years, incomplete questionnaires, and inconsistent responses.

**Figure 1:**
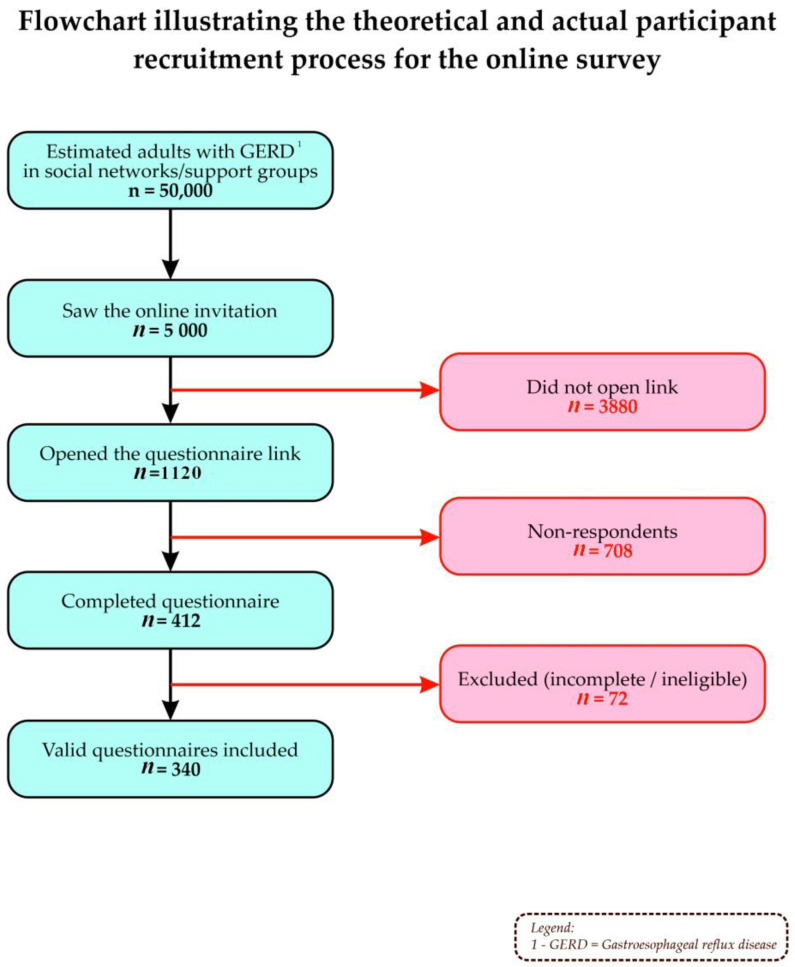
Flow diagram of the participant inclusion process.

The intermediate stages (invitation exposure and link access) represent estimates based on the existing literature regarding response rates in online surveys [14,15], whereas the final stages (number of completed and valid questionnaires) reflect the actual data obtained for the study (Figure 1).

As the recruitment strategy was based on the voluntary participation of people active in online communities, the study sample may include individuals at different stages of disease expression and symptom severity, including both symptomatic and asymptomatic participants despite a prior GERD diagnosis. This heterogeneity in symptom status may have influenced group classification and should be considered when interpreting the findings.

### 2.3. Study Instrument and Pilot Testing

The primary study instrument was the GERD-Q questionnaire, which has been internationally validated for the assessment of typical gastroesophageal reflux symptoms [12]. In this study, the standard GERD-Q items were integrated into an extended questionnaire. The questionnaire comprised 42 items and investigated the following domains: demographic data, nutritional status, typical and associated reflux symptoms, dietary intake of pro- and anti-inflammatory food groups, consumption of potential trigger foods, eating behaviors, and lifestyle-related factors.

The questionnaire was pre-tested on a pilot sample of 30 respondents to assess item clarity. Internal consistency was evaluated using Cronbach’s alpha coefficient (α = 0.87), and inter-item correlation was assessed using Spearman’s correlation coefficient (r = 0.83), indicating good internal reliability. The average time required to complete the questionnaire was approximately 25–30 min.

Although the extended questionnaire used in this study did not undergo formal external validation, the pilot testing and internal consistency indicators suggest that it is adequately reliable for exploratory analyses within an observational study design.

### 2.4. Assessment of GERD Symptoms

The assessment of GERD symptoms included standardized items exploring the frequency and intensity of typical and associated manifestations of gastroesophageal reflux, in accordance with the structure of an internationally validated instrument (GERD-Q) [13].

1. Typical symptoms were evaluated, namely, heartburn (a retrosternal burning sensation) and regurgitation (the sensation of gastric contents returning into the throat or mouth).
2. Associated symptoms were evaluated: epigastric pain; nausea; and reflux-related sleep disturbances, including difficulty falling asleep or nocturnal awakenings caused by symptoms.

Symptom frequency was assessed on a scale ranging from 0 to 4 points (“never” to “4–7 days/week”). Based on these items, the following outcomes were derived: (1) the presence of GERD-type symptoms, defined as the occurrence of typical symptoms at least twice per week, according to the Montreal definition [4], and (2) a symptom severity score, which was obtained by summing the scores for heartburn, regurgitation, epigastric pain, and sleep disturbances (theoretical range: 0–16 points) and subsequently used to classify mild, moderate, and severe symptom forms.

For all comparative analyses and logistic regression models, participants were classified based on their current symptom status using the GERD-Q score (≥8 vs. <8), reflecting the presence or absence of current GERD symptomatology. This cutoff was defined when the questionnaire was first developed and has been shown to provide a good balance between sensitivity and specificity for identifying GERD in clinical and epidemiological settings [13]. The definition of typical symptoms according to the Montreal consensus (symptoms occurring at least twice per week) was used only for descriptive purposes; it was not applied as a classification variable in the statistical analyses.

### 2.5. Sociodemographic, Anthropometric, and Lifestyle Variables

The questionnaire collected the following data:

(a) Demographic characteristics—age, sex, and area of residence (urban/rural)—were collected.
(b) Information on nutritional status—assessed using body mass index (BMI), which was calculated as weight divided by height squared (kg/m^2^)—was collected. Participants’ BMI values were calculated based on self-reported data, and the study population was categorized according to the World Health Organization (WHO) classification [15] as follows: (1) underweight (<18.5 kg/m^2^); (2) normal weight (18.5–24.9 kg/m^2^); (3) overweight (25.0–29.9 kg/m^2^); (4) obesity class I (30.0–34.9 kg/m^2^); (5) obesity class II (35.0–39.9 kg/m^2^); and (6) obesity class III (≥40.0 kg/m^2^).
(c) Lifestyle-related behaviors were collected. The questionnaire included a set of items addressing daily behaviors known to influence GERD symptomatology: (1) smoking status (non-smoker, occasional smoker, and daily smoker, with subcategories based on the average number of cigarettes smoked per day); (2) number of main meals and snacks consumed per day; (3) preferred method of food preparation (home-cooked meals, semi-prepared foods, or fast food); (4) meal timing, including the timing of the last meal relative to bedtime and the frequency of consuming large evening meals (particular attention was paid to adherence to ensuring there is a ≥2 h interval between dinner and bedtime); and (5) usual sleeping position (left/right lateral decubitus, supine, prone, or with the upper body elevated), considering existing evidence on the influence of sleep position on nocturnal reflux.

The assessment of these variables enabled the identification of potentially modifiable behavioral factors associated with the onset or exacerbation of GERD symptoms.

For statistical analysis, several variables were recoded based on the existing literature and clinical relevance. Alcohol and coffee consumption were dichotomized as <3 times/week versus ≥3 times/week. Reflux-related sleep disturbances were recoded into two categories (absent—never/rarely; present—sometimes/often/very often). Sleeping position was classified as reflux-favorable (left lateral decubitus or with a mildly elevated upper body) or reflux-unfavorable (supine, prone, or right lateral decubitus). The frequency of large evening meals was recoded as the frequency of consuming non-large meals (never/rarely) and large meals (frequently/always). The interval between the last meal and sleep was categorized as ≥2 h versus <2 h. Additionally, a composite variable with four categories was generated to assess the cumulative effect of meal volume and timing (non-large + ≥2 h; non-large + <2 h; large + ≥2 h; and large + <2 h).

### 2.6. Dietary Assessment and Food Frequency Recoding

Dietary intake assessment included items designed to investigate the consumption of various food groups with pro- and anti-inflammatory potential. Responses were initially coded on an ordinal scale ranging from 0 to 9 according to consumption frequency (from “never” to “six or more times per day”), allowing for subsequent quantification of the dietary pro- and anti-inflammatory load scores.

For dietary intake analysis, the original 10-point frequency scale was aggregated into a 5-level ordinal scale: 1 = never; 2 = exceptional (occasional); 3 = 1–2 times per week; 4 = 3–4 times per week; and 5 = daily. This recoding was applied to prevent imbalance in the response distribution across categories, improve the stability of statistical models (correlation analyses and multivariable regression), and facilitate epidemiological interpretation of the data without loss of clinically relevant information (Table 1).

The recoded scores were subsequently used in correlational and multivariable analyses to evaluate the relationship between dietary profiles and the severity of gastroesophageal reflux disease (GERD) symptoms.

### 2.7. Construction of Dietary Scores

Food groups with a documented potential to trigger or exacerbate reflux symptoms were investigated based on evidence from the literature. These food groups included high-fat foods (animal fats, fried foods, and fast food), chocolate, coffee and other caffeinated beverages, carbonated drinks, alcoholic beverages, citrus fruits, tomatoes and tomato-based products, and spicy foods.

In addition, the questionnaire included a distinct set of items addressing the consumption of foods with potential anti-inflammatory properties: fresh fruits (excluding citrus fruits), vegetables (excluding tomatoes), fish (including omega-3–rich species such as salmon, mackerel, and sardines), fermented dairy products (yogurt, kefir, and cultured milk products), whole grains, and vegetable oils.

These food groups were selected based on evidence from recent studies linking a higher intake of fiber, antioxidants, polyunsaturated fatty acids, and polyphenols to reductions in oxidative stress and systemic inflammation [11,16].

These dietary scores were constructed specifically for this study based on evidence from the literature regarding foods with pro- and anti-inflammatory potential, and they were used as exploratory indicators of overall dietary patterns.

The PRO and ANTI scores used in this study are not previously validated indices; they were developed specifically for this study based on evidence from the literature regarding foods with pro- and anti-inflammatory or reflux-related potential. Each food group was given equal weight to reflect the cumulative contribution of the overall dietary pattern rather than the isolated effects of individual foods. The threshold of ≥3 times per week was chosen to capture habitual consumption, in line with frequency cutoffs commonly used in observational nutritional studies.

This approach allowed for the identification of overall dietary patterns and the analysis of foods with documented pathophysiological relevance to gastroesophageal reflux, which were subsequently correlated with reported symptom severity.

#### 2.7.1. Pro-Inflammatory Dietary Score (PRO)

The PRO score was calculated by summing food groups considered potentially pro-inflammatory or reflux-promoting. Each food group contributed one point when the reported consumption frequency was ≥3 times per week. The PRO score had a theoretical range of 0 to 9 points, with higher values indicating a predominantly pro-inflammatory dietary profile. Mint consumption was excluded from the score, as it was considered a functional aggravating factor.

The PRO score values were subsequently categorized as low (0–2 points), moderate (3–5 points), or high (≥6 points).

#### 2.7.2. Anti-Inflammatory Dietary Score (ANTI)

The ANTI score was calculated by summing food groups considered potentially protective. One point was assigned to each food category for consumption ≥ 3 times per week. The ANTI score ranged from 0 to 8 points.

The ANTI score values were categorized as low (0–1 points), moderate (2–4 points), or high (≥5 points).

#### 2.7.3. Combined Dietary Balance Score (PRO–ANTI)

The combined dietary balance score was obtained by subtracting the ANTI score from the PRO score (PRO–ANTI), with higher values indicating a predominantly pro-inflammatory dietary profile. Based on score distribution, the PRO–ANTI score was classified into four categories: predominantly anti-inflammatory (≤−2), balanced (−1 or 0), moderately pro-inflammatory (1–2), and predominantly pro-inflammatory (≥3).

The cutoff of ≥3 times per week was chosen to reflect habitual rather than occasional consumption, an approach that is consistent with frequency thresholds commonly used in observational nutritional epidemiology, which, in turn, are in line with approaches used in previous observational studies assessing associations between diet and GERD symptomatology.

### 2.8. Statistical Analysis

Data were initially entered into a Microsoft Excel 2013 database and subsequently exported for statistical analysis using SPSS version 20 and R version 4.3.3.

The analyses included descriptive statistics to characterize the study sample, comparative tests (Student’s *t* test, χ^2^ test, the Kruskal–Wallis test, and ANOVA) to assess differences between groups, and correlational analyses (Pearson and Spearman), depending on the distribution of the variables.

The normality of quantitative variables (age, weight, height, number of main meals and daily snacks, and frequency of physical activity) was assessed using the Kolmogorov–Smirnov test. To identify independent predictors of GERD symptom presence, multivariable logistic regression models were constructed, including variables selected based on clinical relevance and univariate associations, with adjustment for potential confounders. The threshold for statistical significance was set at *p* < 0.05.

## 3. Results

### 3.1. Sample Characteristics

The results should be interpreted in the context of the study design and potential sources of bias, including selection bias related to online recruitment, self-selection of symptomatic individuals, and the use of self-reported data.

The mean age of the participants was 49.3 ± 18.6 years, with a median age of 48 years. Among women, the mean age was 48.17 ± 18.31 years, while that among men was 52.80 ± 19.04 years.

Of the 340 participants included in the analysis, 246 (72.4%) presented current GERD symptoms (GERD-Q score ≥ 8), whereas 94 (27.6%) had no current symptomatology (GERD-Q score < 8) despite a prior diagnosis of GERD (Table 2).

No statistically significant differences were observed between participants with current GERD symptoms and those without current symptomatology with respect to sex, area of residence, or mean age (all *p*-values > 0.05). The distribution of current GERD symptom status across age groups (<30 years, 30–49 years, ≥50 years) did not reveal any significant associations (*p* = 0.282) (Table 3). These findings indicate that age was not an explanatory factor for current GERD symptomatology in this sample.

### 3.2. Association Between Anthropometric Characteristics and GERD Status

Comparative analysis of anthropometric variables did not reveal significant differences between the participants with and without GERD. Body weight, height, and body mass index (BMI) values were comparable between the two groups (all *p* values > 0.05).

The distribution of participants across BMI categories indicated that nearly half of the respondents were normal weight (48.0%), while the remaining participants were classified as overweight (19.7%) or obese (class I: 14.4%; class II: 17.6%; class III: 0.3%). No underweight participants were identified in the sample.

The χ^2^ test was used to evaluate differences in BMI category distribution between groups. It did not reveal a significant association between BMI category and GERD status (*p* = 0.533).

Odds ratio analysis, using the normal-weight category as the reference, did not identify statistically significant associations between overweight or obesity and the presence of GERD, with confidence intervals including the null value of 1.

### 3.3. Association Between Smoking, Alcohol Consumption, and Coffee Consumption and GERD Status

The distribution of smoking status did not reveal statistically significant differences between participants with current GERD symptoms and those without current symptomatology (*p* = 0.351). To assess the potential effect of daily tobacco exposure, smoking status was recoded into a binary variable comparing daily smokers with non-smokers and occasional smokers. This analysis also did not demonstrate a significant association between daily smoking and current GERD symptomatology (*p* = 0.117).

Regarding alcohol consumption, intake frequency was predominantly never or low within the study sample. Comparative analyses did not indicate significant differences between the participants with current GERD symptoms and those without current symptomatology (*p* = 0.823). After alcohol consumption was recoded using a ≥3 times/week threshold, the proportion of participants with current GERD symptoms was slightly higher among frequent consumers; however, this difference did not reach statistical significance (*p* = 0.431), and odds ratio analysis did not indicate a significant independent association.

Similarly, coffee consumption frequency was not significantly associated with current GERD symptomatology (*p* = 0.367). After coffee intake was recoded (<3 vs. ≥3 times/week), the analysis showed a statistically non-significant trend toward a lower proportion of participants with current GERD symptoms among frequent coffee consumers (OR = 0.62; 95% CI: 0.37–1.03), without reaching the threshold for statistical significance (*p* = 0.077).

Overall, neither smoking nor alcohol or coffee consumption was independently associated with current GERD symptomatology in univariate analyses (Table 4).

### 3.4. Association Between Sleep-Related Factors and GERD Status

The analysis of sleep-related factors did not reveal statistically significant associations with the presence of GERD. Reflux-related sleep disturbances did not differ significantly between participants with and without GERD (*p* = 0.400). After the variable was recoded into a binary form (no sleep disturbances vs. presence of sleep disturbances), GERD prevalence was 76.5% among participants without sleep disturbances and 70.1% among those reporting sleep disturbances; this difference did not reach statistical significance (*p* = 0.233). Odds ratio analysis did not indicate a significant association between sleep disturbances and the presence of GERD (OR = 0.72; 95% CI: 0.43–1.21).

Similarly, sleeping position was not significantly associated with GERD status (*p* = 0.844). After sleeping position was recoded into reflux-favorable positions (left lateral decubitus or mildly elevated upper body) versus reflux-unfavorable positions (supine, prone, or right lateral decubitus), the differences remained non-significant (OR = 0.74; 95% CI: 0.47–1.18; *p* = 0.19).

Overall, neither reported sleep disturbances nor recoded sleeping position were independently associated with the presence of GERD in univariate analyses (Table 5).

### 3.5. Association Between Late Meals and GERD Status

Adherence to a schedule in which there was an interval of at least two hours between the last meal and bedtime was significantly associated with GERD status (*p* = 0.030). The participants who consumed meals less than two hours before going to sleep exhibited a higher prevalence of GERD compared with those who consistently respected this interval.

In the recoded analysis (≥2 h vs. <2 h), late meals were associated with a significantly increased risk of GERD (OR = 2.05; 95% CI: 1.25–3.37; *p* = 0.030) (Table 6).

### 3.6. Association Between Large Evening Meals and GERD Status

The analysis of the frequency distribution of large evening meals did not reveal a statistically significant association with the presence of GERD (*p* = 0.698). After we recoded the variable into a binary form (large evening meals reported frequently/always vs. rarely/never), the association remained non-significant, and odds ratio analysis did not indicate an increased likelihood of GERD among participants who reported consuming large evening meals (OR = 1.25; 95% CI: 0.77–2.02; *p* = 0.35).

### 3.7. Cumulative Impact of Meal Volume and Late Meal Timing on GERD

The combined analysis of meal volume and timing of the last meal before bedtime demonstrated a statistically significant association with the presence of GERD (*p* = 0.030).

Participants who reported eating large meals less than two hours before bedtime exhibited the highest prevalence of GERD (80.2%), with a significantly increased risk compared with the reference group (OR = 2.16; 95% CI: 1.18–3.97; *p* = 0.012) (Table 7).

### 3.8. Association Between Individual Food Consumption and GERD (Univariate Analysis)

The univariate analysis of associations between frequent food consumption (≥3 times/week) and the presence of GERD included food groups considered protective/anti-inflammatory and those regarded as aggravating factors for gastroesophageal reflux.

#### 3.8.1. Foods Considered Protective/Anti-Reflux

Among the foods considered to have potential protective effects, the analysis included plant-based milk alternatives (soy, almonds, and rice), fermented dairy products (yogurt and kefir), white meat (chicken and turkey), whole grains (whole-grain bread, brown rice, and whole-grain pasta), non-acidic vegetables, non-acidic fruits (apples and bananas), and unsaturated vegetable oils (olive and canola oil). For these food groups, the distribution of frequent consumption was similar between participants with and without GERD, and no statistically significant associations were identified (all *p* values were >0.05).

In contrast, frequent fish consumption was significantly associated with a lower likelihood of GERD, indicating a potential protective effect (OR = 0.61; 95% CI: 0.38–0.99; *p* = 0.040).

#### 3.8.2. Foods Considered Aggravating/Pro-Reflux

The category of pro-reflux foods included high-fat dairy products, red meat, processed meats, eggs, animal fats (butter and lard), refined sweets, fast food and semi-prepared products, tomatoes and tomato-based products, citrus fruits, spicy foods, coffee, carbonated beverages, and alcohol.

Among these foods, frequent consumption of spicy foods and carbonated beverages was significantly associated with an increased likelihood of GERD (spicy foods: OR = 2.38; 95% CI: 1.01–5.58; *p* = 0.040; carbonated beverages: OR = 2.02; 95% CI: 1.02–3.98; *p* = 0.030). Consumption of tomatoes and tomato-based products showed a trend toward an association with GERD, without reaching statistical significance (OR = 1.69; 95% CI: 0.97–2.95; *p* = 0.056).

For the remaining food groups in this category (high-fat dairy products, red meat, processed meats, eggs, animal fats, refined sweets, fast food/semi-prepared products, citrus fruits, coffee, and alcohol), no statistically significant associations with GERD were identified (all *p* values > 0.05).

The statistically significant findings and observed trends identified in the univariate analysis are summarized in Table 8. These results are based on univariate analyses and should be interpreted with caution, as they do not fully reflect the results of multivariable analyses and do not account for potential confounding factors.

### 3.9. Association Between Current GERD Symptomatology and Pro- and Anti-Inflammatory Dietary Scores

The distribution of the composite dietary scores (PRO, ANTI, and PRO–ANTI) within the study sample is presented below. Dietary scores were analyzed using categorized levels reflecting low, moderate, and high nutritional exposure.

The PRO score was recoded into three nutritional categories. The majority of participants presented a moderate PRO score (65.0%), while 21.5% had a high PRO score ≥ 6 pro-inflammatory food items consumed frequently), and 13.5% had a low PRO score.

Regarding the ANTI score, most participants (75.6%) were classified within the moderate category, whereas 13.5% presented a high ANTI score, and 10.9% had a low ANTI score.

The dietary balance score (PRO–ANTI) was recoded into four categories to describe the overall dietary patterns. The participant distribution was as follows: 14.1% had a predominantly anti-inflammatory profile (≤−2), 25.6% had a relatively balanced profile (−1 to 0), 35.6% had a moderately pro-inflammatory profile (1–2), and 24.7% had a predominantly pro-inflammatory profile (≥3).

The association between overall dietary patterns and current GERD symptomatology was subsequently evaluated using multivariable logistic regression models, adjusted for demographic, anthropometric, and behavioral factors.

The associations between composite dietary scores and current GERD symptomatology are summarized in Table 9.

A statistically significant association was identified between categories of the pro-inflammatory dietary score (PRO) and current GERD symptomatology (*p* = 0.048). The proportion of participants with a high PRO score (≥6) was greater among participants with current GERD symptoms compared with those without current symptomatology (12.2% vs. 5.3%). Compared with the low-PRO-score category, a high PRO score was associated with an approximately threefold-higher likelihood of current GERD symptomatology (OR = 2.9).

In contrast, the anti-inflammatory dietary score (ANTI) was not significantly associated with current GERD symptomatology (*p* = 0.739), with a similar distribution of categories among participants with and without current symptomatology.

Analysis of the combined PRO–ANTI score did not reveal a significant overall association with current GERD symptomatology (*p* = 0.775). Although the proportion of participants classified as having a predominantly pro-inflammatory profile was higher among participants with current GERD symptoms compared with those without current symptomatology (43.5% vs. 37.2%), this difference did not reach statistical significance.

Given the significant association observed for the PRO score, composite dietary scores were subsequently included in multivariable logistic regression models to evaluate the independent relationship between overall dietary patterns and current GERD symptomatology, after adjustment for potential demographic, anthropometric, and lifestyle-related confounders.

### 3.10. Multivariable Analysis of Factors Associated with GERD

The variables that showed statistically significant associations or consistent trends in univariate analyses were included in multivariable logistic regression models to evaluate their independent relationships with the presence of gastroesophageal reflux disease (GERD).

In the dietary model based on the pro-inflammatory dietary score (PRO), the PRO score was independently and consistently associated with the presence of GERD (Table 10). Compared with a low PRO score, a moderate PRO score was associated with a significantly increased risk of GERD both in the dietary model adjusted for age and sex (OR = 2.12; 95% CI: 1.15–3.89; *p* = 0.016) and in the integrated dietary and behavioral model (OR = 2.01; 95% CI: 1.08–3.73; *p* = 0.027). A high PRO score was associated with an even greater risk of GERD—exceeding a fourfold increase—in both the dietary model (OR = 4.67; 95% CI: 1.58–13.78; *p* = 0.005) and the integrated model (OR = 4.23; 95% CI: 1.42–12.62; *p* = 0.009).

Age emerged as an independent predictor of GERD in all of the models analyzed, with each additional year being associated with a modest but statistically significant increase in GERD risk (with ORs ranging between 1.02 and 1.03, and all *p* values were < 0.05). Sex, body mass index (BMI), and the anti-inflammatory dietary score (ANTI) were not significantly associated with the presence of GERD in the PRO-based models.

## 4. Discussion

This study evaluated the relationship between dietary patterns and pro- and anti-inflammatory potential, lifestyle-related behaviors, and the presence of gastroesophageal reflux symptoms in an adult population from Romania.

The main findings indicate that a predominantly pro-inflammatory dietary profile and late timing of the last meal before bedtime are significantly associated with current GERD symptomatology, including after adjustment for age, sex, body mass index, and other behavioral factors. Although age did not significantly differentiate participants with current GERD symptoms and those without current symptomatology in descriptive analyses, it emerged as a modest but statistically significant predictor of current GERD symptomatology in multivariable models, suggesting a cumulative effect in interaction with dietary and behavioral factors. In this context, other factors, particularly dietary patterns and lifestyle-related behaviors, may play a more relevant role than age alone in explaining the presence of symptoms of gastroesophageal reflux disease (GERD) in this population.

In contrast, most foods considered protective and several traditional lifestyle-related factors (smoking, alcohol consumption, sleeping position, and large evening meals, when analyzed in isolation) did not show independent significant associations with GERD symptomatology.

The high prevalence of symptoms compatible with GERD observed in this study should be interpreted in light of the online recruitment strategy employed, which may have preferentially attracted individuals experiencing symptoms. As such, this proportion should not be regarded as a population-level prevalence estimate. Furthermore, the imbalance between participants with active symptoms and those without symptoms at the time of assessment may have affected the precision of some estimates and limited our ability to detect weaker associations. This imbalance may also have contributed to wider confidence intervals and reduced statistical power, particularly for variables with small effect sizes.

### 4.1. Association Between Pro-Inflammatory Dietary Patterns and the Presence of GERD

The most consistent finding of this study was the significant association between the pro-inflammatory dietary score and the presence of GERD. Both univariate analyses and multivariable logistic regression models revealed a progressive increase in the risk of GERD with an increase in PRO score, with participants in the high-PRO category exhibiting an over fourfold-higher risk compared with those with a low PRO score. This finding suggests that in the studied sample, GERD is more strongly associated with cumulative exposure to multiple food groups with irritant or inflammatory potential than to the consumption of a single food item in isolation [17].

However, these scores should be interpreted as exploratory proxies for overall dietary patterns rather than standardized clinical indices.

This observation is consistent with the recent literature, which has increasingly moved away from a focus on isolated “trigger foods” toward the concept of overall dietary patterns. Several studies and literature syntheses indicate that diets rich in fats (particularly saturated fats) ultra-processed foods, and refined sugars are associated with a higher frequency of reflux symptoms and/or unfavorable physiological parameters [18,19,20,21]. For example, El-Serag et al. reported associations between fat intake and the presence of reflux symptoms in an observational study [19]. Similarly, Song et al. demonstrated that specific dietary patterns and eating behaviors are associated with the presence of GERD symptoms [20].

From a pathophysiological perspective, such dietary patterns may promote reflux through multiple mechanisms, including gastric distension, alterations in the frequency of transient lower-esophageal-sphincter relaxation, and increased esophageal acid exposure—mechanisms discussed in physiological studies and reviews of lifestyle interventions [18,22,23]. Low-grade systemic inflammation represents a plausible mechanism for symptom amplification and has been discussed in the context of dietary inflammatory indices [11]. Supporting this hypothesis, recent cohort data indicate that a higher pro-inflammatory dietary potential is associated with an increased risk of GERD [24]. In agreement with these findings, recent prospective studies evaluating global dietary patterns have reported a lower risk of GERD among individuals adhering to predominantly vegetarian diets compared with those with non-vegetarian dietary patterns [25].

The lack of an independent association between the ANTI score and GERD, alongside the persistent predictive value of the PRO score, suggests that the cumulative effects of harmful foods may outweigh the benefits of protective foods when both coexist within a mixed dietary pattern. This may explain the loss of statistical significance observed for the combined PRO–ANTI score in certain analyses.

Although some foods with potential protective effects, such as fish, demonstrated favorable associations in univariate analyses, these effects were insufficient to counterbalance the overall impact of frequent consumption of irritant, highly processed, or high-fat foods. This finding is consistent with the hypothesis that nutritional strategies for GERD should focus not only on the “addition” of healthy foods but also on a systematic reduction in the consumption of foods with pro-reflux potential.

Similar results have been reported in studies showing that simply increasing fruit and vegetable intake is insufficient to alleviate symptoms if one’s overall diet remains rich in fats and ultra-processed products [21,26,27].

These findings suggest that the role of individual food items should be interpreted within the broader context of overall dietary patterns.

### 4.2. Specific Role of Spicy Foods and Carbonated Beverages

Among the individual food items, spicy foods and carbonated beverages were the only ones significantly associated with GERD in the univariate analysis. Frequent consumption of these items was associated with an approximately twofold-higher risk of GERD. These findings are consistent with experimental data demonstrating that chemical stimulation of the esophagus with capsaicin can increase esophageal sensitivity and pain perception [28]. In addition, physiological studies have shown that carbonated beverages may induce gastric distension and reduce lower-esophageal-sphincter pressure [29]. A systematic review of the literature also supports the existence of an association between carbonated-beverage consumption and reflux symptoms [30].

However, the loss of statistical significance in the multivariable models suggests that these foods may be markers of an overall dietary pattern rather than independent risk factors. This finding highlights the importance of distinguishing between univariate associations and independent predictors identified in multivariable models. This interpretation is supported by the persistence of the significance of behavioral factors related to meal timing after adjustment, highlighting the value of composite dietary scores. These results are in line with conclusions from previous studies emphasizing that the effects of diets on GERD are rarely attributable to a single food item [18,26].

### 4.3. Importance of Meal Timing and Cumulative Effect of Eating Behaviors

Another central finding of this study is the consistent association between late meals (consumption of the last meal of the day less than two hours before bedtime) and the presence of GERD. This association was significant in both univariate analyses and multivariable models, and it was further reinforced by the combined analysis, which indicated that there was a higher likelihood of GERD among participants who reported both large evening meals and late meal timing. In line with these findings, the literature supports the role of the dinner-to-bedtime interval: a shorter interval between dinner and sleep was associated with a higher risk of GERD in a case–control study [31]. Moreover, reviews focusing on lifestyle interventions include avoidance of late meals among measures associated with symptom improvement, particularly nocturnal reflux [23].

Kaltenbach et al., in the American College of Gastroenterology (ACG) guidelines, identify the avoidance of late meals as one of the few behavioral interventions supported by solid physiological evidence [31]. Similarly, Fujiwara and Arakawa demonstrated that the supine position combined with increased gastric contents may promote reflux through loss of the gravitational effect and reduced esophageal clearance [32].

The combined analysis in this study indicates that GERD symptoms are most likely to occur when late meals are combined with a large meal volume, suggesting a cumulative effect of food volume and a short interval between the last meal and bedtime. This observation is supported by research showing that a reduced dinner-to-bedtime interval is associated with a higher frequency of reflux episodes, particularly nocturnal reflux, as well as by experimental and clinical studies indicating that large-volume meals may increase intragastric pressure and exposure to esophageal acid. Recent reviews emphasize the role of evening eating habits, including late meals and overeating, as modifiable risk factors for GERD symptomatology [18,33,34]. Taken together, these findings emphasize the interplay between meal timing and meal volume in regard to reflux risk.

The observed association for the combined exposure (large meals and late timing) likely reflects a cumulative physiological effect, in which increased gastric volume and a shorter interval before lying down act together to promote reflux. This may explain why each factor alone did not consistently reach statistical significance, while their combination showed a stronger and more consistent association.

The finding that large evening meals analyzed in isolation were not significantly associated with GERD but became relevant when combined with late meal timing suggests that meal volume and timing have a synergistic effect. This observation has relevant clinical implications. Synchronization of meals with the sleep–wake cycle appears to be potentially more relevant than an exclusive focus on meal quantity or strict avoidance of individual foods.

From a practical perspective, these findings support the notion that avoiding the consumption of meals less than two hours before bedtime and reducing the size of evening meals are simple and easily applicable ways of alleviating reflux symptoms.

### 4.4. Classical Lifestyle Factors: Smoking, Alcohol Consumption, Coffee Consumption, and Sleeping Position

The lack of independent associations for smoking, alcohol consumption, coffee consumption, and sleeping position is consistent with the contemporary literature, which reports heterogeneous and often inconsistent findings. Conflicting results regarding smoking and alcohol are also reflected in recent evidence. Population-based cohort data suggest that the cessation of smoking may be associated with an improvement in reflux symptoms [35]. However, the absence of a significant association between smoking and GERD in this study does not exclude a potential clinical relationship, but it may reflect limitations related to sample size, exposure variability, or behavioral adaptations among participants. In contrast, meta-analyses addressing alcohol consumption indicate that there is a heterogeneous relationship, with substantial variability across studies [36].

The observed lack of statistically significant associations may reflect several factors, including the relatively homogeneous distribution of these behaviors within the sample, pre-existing behavioral adaptations among symptomatic individuals (e.g., reduced alcohol intake), and the inherent limitations of self-reported measurements. Collectively, these findings suggest that in the current context, dietary patterns and meal-related behaviors may exert a more direct impact on reflux symptoms than certain classical lifestyle factors considered in isolation.

Similarly, coffee consumption was not significantly associated with GERD in this study. A non-significant trend toward a lower prevalence of GERD among frequent coffee consumers was observed; however, this finding may reflect residual confounding or behavioral adaptations among symptomatic individuals rather than a true protective effect.

With respect to sleeping position, recent data indicate that left lateral decubitus is associated with reduced nocturnal acid exposure and improved esophageal clearance [37], findings that are also supported by a recent systematic review [38]. Our results suggest that the effect of sleeping position may be outweighed by other dietary and behavioral factors under real-life conditions. This difference may be explained by the more direct physiological impact of meal timing on reflux, whereas the effects of sleeping position may be more subtle or influenced by behavioral variability and self-reported measures.

These findings are consistent with the notion that dietary patterns play a broader role in shaping GERD risk.

### 4.5. The Combined PRO–ANTI Score and Nutritional Coherence of the Findings

Although the PRO–ANTI score was not significantly associated with GERD in univariate analysis, examination of the combined PRO–ANTI score revealed a directional trend toward a higher prevalence of GERD among participants with a predominantly pro-inflammatory dietary profile. In the alternative multivariable model, this category was associated with a significantly increased risk of GERD, suggesting that global dietary imbalance becomes relevant after adjustment for behavioral and demographic factors. This approach is aligned with studies in which global dietary indices were employed to assess the impact of diet on inflammatory conditions [11,24].

These findings support the hypothesis that an overall dietary imbalance may be more relevant than the isolated consumption of individual “healthy” or “unhealthy” foods. In this context, dietary interventions may aim to reconfigure the overall dietary pattern rather than focusing solely on the elimination of foods perceived as “triggers” in isolation.

### 4.6. Clinical and Nutritional Implications

Our findings indicate the prudence of integrating global dietary pattern assessment into GERD management, as proposed in modern approaches to treating the condition [18,21,26].

This perspective is consistent with clinical guideline recommendations, which emphasize the multifactorial nature of GERD and the importance of considering behavioral and lifestyle-related factors in symptom management [9].

Future nutritional interventions may focus on reducing overall dietary inflammatory load and optimizing meal timing rather than targeting individual foods in isolation [23,26].

The lack of an independent association between BMI and GERD suggests that the relationship between excess body weight and reflux may be partially mediated by dietary and lifestyle behaviors. This observation is supported by population-based and interventional studies indicating that behavioral modifications may improve symptoms even independently of weight loss [23].

From a public health perspective, these results highlight the importance of simple nutritional and behavioral interventions—such as optimizing dietary patterns and avoiding late meals—as potentially relevant strategies for reducing the burden of gastroesophageal reflux symptoms in the adult population. Future studies incorporating longitudinal designs, interaction analyses, and more comprehensive multivariable models may foster a deeper understanding of causal relationships.

## 5. Limitations and Strengths

The findings of this study should be interpreted in the context of its design and methodology, which include both inherent limitations and several methodological strengths.

This study’s cross-sectional observational design allows for the identification of associations between dietary patterns, behavioral factors, and the presence of gastroesophageal reflux symptoms, but it does not permit causal inference. Future studies, particularly longitudinal or interventional designs, as well as more comprehensive multivariable models including a broader range of potential confounders, may help better clarify the causal relationships between dietary factors and reflux symptoms. In addition, recruitment conducted exclusively through online channels may introduce self-selection bias, which could partly explain the relatively high prevalence of GERD observed in the sample and may limit the findings’ generalizability with respect to the general population.

Dietary intake, lifestyle behaviors, and symptom data were self-reported and may therefore have been affected by recall bias, reporting bias, and potential misclassification, particularly given the variability in symptom perceptions and the potential presence of silent reflux. Moreover, the absence of objective clinical data (such as endoscopy, pH monitoring, impedance testing, or information on antisecretory therapy use) limits diagnostic confirmation and the differentiation of GERD subtypes.

Dietary assessment was based on the frequency of consumption of selected food items, chosen according to the existing literature on foods with pro- and anti-reflux potential, without quantifying portion sizes, total energy intake, or macronutrient composition. This limits the capacity for detailed nutritional analysis and adjustment for total caloric intake, although it does not affect the evaluation of overall dietary patterns, which represented the primary objective of the study.

In addition, the dietary scores used in this study (PRO and ANTI) were not previously validated instruments but were instead developed specifically for the purposes of this study, based on existing evidence regarding foods with pro- and anti-inflammatory or reflux-related potential. These scores should therefore be interpreted as exploratory indicators of overall dietary patterns. Although their construction was informed by the literature, the lack of external validation may limit comparability with other studies and should be considered when interpreting the findings. In addition, the equally weighted components and predefined frequency thresholds employed may not fully capture the differential effects of individual foods or the complexity of dietary exposures.

The analyses examining the associations between individual food items and GERD were exploratory. Therefore, findings related to individual foods should be interpreted cautiously and in the context of the overall dietary patterns assessed through the composite dietary scores.

Univariate analyses should be interpreted with caution, as they do not control for confounding factors.

Several potentially relevant confounding variables were not systematically assessed, including stress levels, physical activity, digestive comorbidities, and therapeutic history, all of which may influence both dietary behaviors and reflux symptoms.

The lack of an observed association between BMI and GERD in this study may have been influenced by the sample’s characteristics, including limited variability in BMI distribution, potential selection bias, and unmeasured confounding factors.

An additional limitation is the imbalance between participants presenting active GERD symptoms and those without current symptoms at the time of assessment, even though all participants had a prior diagnosis of GERD. This unequal distribution may have affected the statistical power of comparisons and limited the precision of effect estimates. Future studies including a more balanced distribution of participants across different symptom profiles are needed to improve the robustness and generalizability of the findings.

Although comparisons were performed between participants classified based on their current symptom status (i.e., individuals with and without current GERD symptoms despite a prior diagnosis of GERD), the study design remains cross-sectional, and group classification was based on symptom scores rather than predefined sampling strategies; therefore, the results should not be interpreted within a case–control framework.

Nevertheless, these limitations are comparable in type and magnitude to those reported in similar observational nutritional studies [20,21]. At the same time, this study has several notable strengths, including a relatively large sample size, the use of a validated instrument for symptom assessment (GERD-Q), evidence-based selection of food items, the construction of dietary scores reflecting global dietary patterns, the integration of behavioral factors, and the use of multivariable models allowing adjustment for potential confounders.

## 6. Conclusions

This study suggests potential associations between pro-inflammatory dietary patterns, meal timing, and gastroesophageal reflux symptoms; however, these findings should be considered in light of the study design and sampling limitations. In contrast, several classical lifestyle-related factors and most individual foods did not show independent significant associations in this sample, although these findings should be interpreted with caution.

These findings highlight the potential value of an integrated approach to GERD management that focuses on overall dietary patterns and modifiable eating behaviors rather than the strict elimination of individual foods. From a clinical and public health perspective, nutritional strategies aimed at reducing the inflammatory load of a diet and avoiding late meals may contribute to reducing the burden of gastroesophageal reflux symptoms in the adult population, although further studies are needed to confirm these associations.

## Figures and Tables

**Table 1:** Recoding scheme applied to the original 9-point food frequency scale.

Original Frequency (9 Levels)	Initial Code	Recoded Category	New Code (1–5)
Never	0	Never	1
Once in the last month	1	Exceptional	2
2–3 times in the last month	2	Exceptional	2
1–2 times per week	3	1–2 times/week	3
3–4 times per week	4	3–4 times/week	4
5–6 times per week	5	Daily	5
Once per day	6	Daily	5
2–3 times per day	7	Daily	5
≥4–5 times/day	8	Daily	5
≥6 times/day	9	Daily	5

The 9-point frequency scale was collapsed into 5 ordinal categories (1–5) to ensure that there was a balanced distribution across levels and to improve the robustness of the multivariate statistical models.

**Table 2:** Distribution of participants according to current GERD symptom status (GERD-Q ≥ 8 vs. < 8).

GERD Status	n	%
No current GERD symptoms (GERD-Q < 8)	94	27.6
Current GERD symptoms (GERD-Q ≥ 8)	246	72.4
Total	340	100

GERD-Q, Gastroesophageal Reflux Disease Questionnaire. Percentages are expressed as proportions of the total sample.

**Table 3:** Distribution of current GERD symptom status across age groups (GERD-Q ≥ 8 vs. < 8).

Age Group	No Current Symptoms (GERD-Q < 8) (n/%)	Current GERD Symptoms (GERD-Q ≥ 8) (n/%)
<30 years	16 (17.02%)	48 (19.51%)
30–49 years	27 (28.72%)	88 (35.77%)
≥50 years	51 (54.26%)	110 (44.72%)

GERD-Q, Gastroesophageal Reflux Disease Questionnaire. Data are presented as number (percentage).

**Table 4:** Recoded behavioral factors and their associations with current GERD symptomatology (univariate analysis).

Behavioral Factor (Recoded)	No Current Symptoms (GERD-Q < 8) n (%)	Current GERD Symptoms (GERD-Q ≥ 8) n (%)	OR (95% CI)	*p*-Value
Daily smoking vs. non-/occasional smoking	27 (34.6%)	51 (65.4%)	—	0.117
Alcohol consumed ≥3 times/week vs. <3 times/week	4 (20.0%)	16 (80.0%)	1.57 (0.51–4.81)	0.431
Coffee consumed ≥3 times/week vs. <3 times/week	66 (31.1%)	146 (68.9%)	0.62 (0.37–1.03)	0.077

OR, odds ratio; CI, 95% confidence interval; GERD-Q, Gastroesophageal Reflux Disease Questionnaire. *p*-values were derived from the χ^2^ test. Statistical significance was set at *p* < 0.05. OR was not calculated for variables not meeting model assumptions.

**Table 5:** Recoded sleep-related factors and their association with current GERD symptomatology (univariate analysis).

Sleep-Related Factor (Recoded)	No Current Symptoms (GERD-Q < 8) n (%)	Current GERD Symptoms (GERD-Q ≥ 8) n (%)	OR (95% CI)	*p*-Value
Sleep disturbances (yes vs. no)	66 (29.9%)	155 (70.1%)	0.72 (0.43–1.21)	0.233
Unfavorable vs. favorable sleeping position	54 (25.6%)	157 (74.4%)	0.74 (0.47–1.18)	0.19

OR, odds ratio; CI, 95% confidence interval; GERD-Q, Gastroesophageal Reflux Disease Questionnaire. *p*-values were derived from the χ^2^ test. Statistical significance was set at *p* < 0.05.

**Table 6:** Late meals (last meal < 2 h before bedtime) and current GERD symptomatology (recoded).

Meals Before Bedtime (Recoding)	No Current Symptoms (GERD-Q < 8) (n, %)	Current GERD Symptoms (GERD-Q ≥ 8) (n, %)	OR (95% CI)	*p*-Value
0—≥2 h before bedtime (always)	52 (34.9%)	97 (65.1%)	Ref.	–
1—Late meals (<2 h before bedtime) (frequent or occasional)	42 (22.0%)	149 (78.0%)	2.05 (1.25–3.37)	0.030

χ^2^(1) = 7.04, *p* = 0.030. OR, odds ratio; CI, 95% confidence interval; GERD-Q, Gastroesophageal Reflux Disease Questionnaire. Statistical significance was set at *p* < 0.05.

**Table 7:** Combined analysis of large evening meals and late meal timing in relation to current GERD symptomatology.

Combined Behavioral Group	No Current Symptoms (GERD-Q < 8) (n, %)	Current GERD Symptoms (GERD-Q ≥ 8) (n, %)	OR (95% CI)	*p*-Value
G1—Non-large meal + ≥2 h before bedtime	36 (32.4%)	75 (67.6%)	Ref.	–
G2—Non-large meal + <2 h before bedtime	19 (25.3%)	56 (74.7%)	1.39 (0.74–2.60)	0.29
G3—Large meal + ≥2 h before bedtime	16 (42.1%)	22 (57.9%)	0.64 (0.32–1.26)	0.20
G4—Large meal + <2 h before bedtime	23 (19.8%)	93 (80.2%)	2.16 (1.18–3.97)	0.012

Global χ^2^(3) = 8.91, *p* = 0.030. OR, odds ratio; CI, 95% confidence interval; GERD-Q, Gastroesophageal Reflux Disease Questionnaire. Reference category: G1 (non-large meal + ≥2 h before bedtime). Statistical significance was set at *p* < 0.05. Large meal was defined as a self-reported large portion size at the last meal of the day.

**Table 8:** Individual food items associated with current GERD symptomatology (univariate analysis, ≥3 times/week vs. <3 times/week).

Food Item (Exposure ≥ 3 Times/Week)	Direction of Association with GERD Symptoms	OR (95% CI)	*p*-Value
Fish	Protective	0.61 (0.38–0.99)	0.040
Spicy foods	Increased risk	2.38 (1.01–5.58)	0.040
Carbonated beverages	Increased risk	2.02 (1.02–3.98)	0.030
Tomatoes/tomato products (borderline significance)	Increased risk	1.69 (0.97–2.95)	0.056

OR, odds ratio; CI, 95% confidence interval; GERD, Gastroesophageal Reflux Disease. *p*-values were derived from univariate logistic regression analysis. Statistical significance was set at *p* < 0.05.

**Table 9:** Association between current GERD symptomatology and dietary scores.

Dietary Score	Category	No Current Symptoms (GERD-Q < 8) (n, %)	Current GERD Symptoms (GERD-Q ≥ 8) (n, %)	χ^2^	*p*-Value
PRO	Low (0–2)	20 (21.3%)	68 (27.6%)		
	Moderate (3–5)	69 (73.4%)	148 (60.2%)		
	High (≥6)	5 (5.3%)	30 (12.2%)	6.14	0.048
ANTI	Low (0–1)	49 (52.1%)	135 (54.9%)		
	≥2	45 (47.9%)	111 (45.1%)	0.11	0.739
PRO–ANTI	≤−2	4 (4.3%)	10 (4.1%)		
	−1/0	18 (19.1%)	42 (17.1%)		
	1–2	37 (39.4%)	87 (35.4%)		
	≥3	35 (37.2%)	107 (43.5%)	1.19	0.775

PRO, pro-inflammatory dietary score; ANTI, anti-inflammatory dietary score; PRO–ANTI, dietary balance score (PRO minus ANTI); GERD-Q, Gastroesophageal Reflux Disease Questionnaire. Data are presented as number (percentage). χ^2^ and *p*-values refer to the overall comparison across categories within each dietary score. Statistical significance was set at *p* < 0.05.

**Table 10:** Multivariable logistic regression models examining the association between the pro-inflammatory dietary score (PRO) and current GERD symptomatology.

Predictor	Dietary Model (PRO-Based) OR (95% CI)	*p*-Value	Integrated Model (Adjusted) OR (95% CI)	*p*-Value
Moderate vs. low PRO scores	2.12 (1.15–3.89)	0.016	2.01 (1.08–3.73)	0.027
High vs. low PRO scores	4.67 (1.58–13.78)	0.005	4.23 (1.42–12.62)	0.009
Moderate vs. low ANTI scores	0.89 (0.55–1.45)	0.649	—	—
Late meals (<2 h before bedtime vs. ≥2 h)	—	—	1.65 (0.97–2.81)	0.066
Consumption of spicy foods (≥3 times/week)	—	—	1.54 (0.92–2.58)	0.102
Consumption of carbonated beverages (≥3 times/week)	—	—	1.42 (0.85–2.37)	0.182
BMI	—	—	1.03 (0.99–1.07)	0.123
Age (years)	1.02 (1.00–1.04)	0.038	1.02 (1.00–1.04)	0.045
Sex (male vs. female)	1.34 (0.82–2.19)	0.245	1.41 (0.85–2.34)	0.184

PRO, pro-inflammatory dietary score; ANTI, anti-inflammatory dietary score; OR, odds ratio; CI, 95% confidence interval; BMI, body mass index. The integrated model was adjusted for age, sex, BMI, dietary factors, and lifestyle variables. Reference category: low PRO score. Statistical significance was set at *p* < 0.05.

## Data Availability

The data are not publicly available due to privacy and ethical restrictions. Aggregated results are available from the corresponding author upon reasonable request.

## Abbreviations

The following abbreviations are used in this manuscript:

ANTI	Anti-inflammatory dietary score
BMI	Body mass index
CI	Confidence interval
GERD	Gastroesophageal reflux disease
GERD-Q	Gastroesophageal Reflux Disease Questionnaire
OR	Odds ratio
PRO	Pro-inflammatory dietary score
PRO–ANTI	Combined dietary balance score
